# Correction: Bibi et al. Valorization of Agricultural Waste as a Chemiresistor H_2_S-Gas Sensor: A Composite of Biodegradable-Electroactive Polyurethane-Urea and Activated-Carbon Composite Derived from Coconut-Shell Waste. *Polymers* 2023, *15*, 685

**DOI:** 10.3390/polym16172504

**Published:** 2024-09-03

**Authors:** Aamna Bibi, Karen S. Santiago, Jui-Ming Yeh, Hsiu-Hui Chen

**Affiliations:** 1 Department of Chemistry, Center for Nanotechnology and R & D Center for Membrane Technology at Chung Yuan Christian University, Chung Li 32023, Taiwan; emi2118@gmail.com; 2 Department of Chemistry, College of Science, Research Center for the Natural and Applied Sciences, University of Santo Tomas, Manila 1015, Philippines; kssantiago@ust.edu.ph; 3 Department of Molecular Science and Engineering, National Taipei University of Technology, Taipei 10608, Taiwan

References [42,43] have been removed as they have been identified as being irrelevant to our study due to a scope mismatch. With this correction, the order of some references has been adjusted accordingly. The authors state that the scientific conclusions are unaffected. This correction was approved by the Academic Editor. The original publication has also been updated [1].

[42] Bahadur, A.; Iqbal, S.; Alsaab, H.O.; Awwad, N.S.; Ibrahium, H.A. Designing a novel visible-light-driven heterostructure Ni–ZnO/S-g-C_3_N_4_ photocatalyst for coloured pollutant degradation. *RSC Adv.* **2021**, *11*, 36518–36527.
[43] Iqbal, S.; Bahadur, A.; Javed, M.; Liu, G.; Al-Muhimeed, T.I.; AlObaid, A.A.; Ahmad, Z.; Feng, K.; Xiao, D. Synergetic intimate interface contacts of 2D/1D S-g-C_3_N_4_/Co-NiS heterojunction with spatial charge separation for boosting photodegradation of MB and inactivation of pathogens under visible light irradiation. *J. Alloys Compd.* **2021**, *892*, 162012.
